# Estrogen Formation and Inactivation Following TBI: What we Know and Where we Could go

**DOI:** 10.3389/fendo.2020.00345

**Published:** 2020-05-29

**Authors:** Kelli A. Duncan

**Affiliations:** Department of Biology and Program in Neuroscience and Behavior, Vassar College, Poughkeepsie, NY, United States

**Keywords:** TBI, estrogen, aromatase, androgen, sulfatase, HSD17B

## Abstract

Traumatic brain injury (TBI) is responsible for various neuronal and cognitive deficits as well as psychosocial dysfunction. Characterized by damage inducing neuroinflammation, this response can cause an acute secondary injury that leads to widespread neurodegeneration and loss of neurological function. Estrogens decrease injury induced neuroinflammation and increase cell survival and neuroprotection and thus are a potential target for use following TBI. While much is known about the role of estrogens as a neuroprotective agent following TBI, less is known regarding their formation and inactivation following damage to the brain. Specifically, very little is known surrounding the majority of enzymes responsible for the production of estrogens. These estrogen metabolizing enzymes (EME) include aromatase, steroid sulfatase (STS), estrogen sulfotransferase (EST/SULT1E1), and some forms of 17β-hydroxysteroid dehydrogenase (HSD17B) and are involved in both the initial conversion and interconversion of estrogens from precursors. This article will review and offer new prospective and ideas on the expression of EMEs following TBI.

## Introduction

Traumatic brain injury (TBI) is a leading cause of human death and morbidity worldwide. TBI is a broad term to explain any injury or damage to the central nervous system, ranging from concussive, penetrating, to ischemic stroke. Over 70,000 cases are reported a year and it affects millions of people in the United States alone (1, 2). At-risk groups range from children, who frequently participate in contact sports to military personnel, and to an increasingly active elderly population (3, 4). Disturbingly high injury rates have made TBI not only an issue of social and economic concern, but also the target of nearly 50 years of dedicated clinical research (5–7). Despite research and improvements in effective patient care, there still remain very few recommended treatments which leads to poor patient outcomes (8).

Injury to the brain occurs in two phases, regardless of the cause. The irreversible primary phase is the injury itself. The majority of research around the primary phase focuses on decreasing the chance of injury (e.g., helmet design). The secondary and potentially reversible phase, which begins after the initial injury and continues for days to weeks afterword, is characterized by an induction of a neuroinflammatory response. This response is characterized by a permeation of inflammatory cells around the injury, followed by endothelial activation, and an accumulation of inflammatory cytokines (9–11). This secondary phase has some beneficial effects, but commonly results in an exacerbation of deleterious inflammatory effects, decreased cognitive ability, motor loss, and increased risk of neurodegenerative diseases, such as multiple sclerosis (12–17). This secondary response to injury is well-characterized across vertebrates (rodents, birds, fish) (18–20). The majority of research has focused on preventing this secondary response (21, 22). One pharmacotherapeutic strategy for treatment of secondary injury has revolved around the role of sex steroids, specifically estrogens on the attenuation of the immune response (22–25). Briefly, we will review the effects of estrogens on cell survival and neuroinflammation following TBI.

Following damage to the brain, estrogens can promote neurogenesis and neural recovery by attenuating neural outgrowth and glial activity (25–29). Estrogens are also indicated to serve as an antioxidant against intrinsic free radical production following TBI (30, 31). The majority of these effects are through estrogen receptor (ER) signaling pathways. The genomic signal transduction pathway of estrogens involves the dimerization of ERs and transcriptional regulation of estrogen mediated target genes which in turn can provide neuroprotective effects, evidenced by decreased inflammation, reactive gliosis, and edema (30, 32–34). Furthermore, activation of ER alpha, the primary mediator of steroid induced neuroprotection, can also alter neurovascular function and promote myelin repair (24, 35). However, rapid non-genomic signaling has been shown to upregulate estrogen mediated neuroprotection via the pro-survival signaling pathway, phosphoinositide 3-kinase/Akt. Activation of this pathway results in increased cell survival, differentiation, and growth (36). Estrogens can also mediate cell death and apoptosis by inhibiting subcellular trafficking of p38α. Jun N-terminal kinase (JNK) and p38 are necessary for the activation of pro-apoptotic signaling pathways (34, 36, 37). Moreover, when estrogens are given or produced immediately after injury, they can decrease pro-inflammatory and increase anti-inflammatory cytokines and thus have strong antioxidant and anti-inflammatory effects. In terms of behavior and cognitive ability following TBI, very little is known about the role of estrogens in ameliorating these symptoms. However, estrogens can also enhance memory function and neurological outcome in male rodents following TBI (38–47). In addition to these effects on cell/neuronal survival, estrogens can also regulate neuroinflammation following injury.

Estrogen synthesis following TBI reduces the concentration of pro-inflammatory cytokines and thus decreases the secondary wave of degeneration observed following damage to the brain (30, 48–50). Through regulation of pro-inflammatory cytokines, estrogens subsequently reduce leukocyte recruitment, cerebral edema, apoptosis, and reactive astrogliosis, thusly improving outcomes following injury (51). Thus, estrogens are neuroprotective on many fronts following TBI (52, 53). While, I've only touched on a few of the pre-clinical studies, the majority of these studies suggest that estrogens are neuroprotective following TBI. However, clinical trials in humans involving estrogen administration have not shown as robust of a beneficial result, thus identifying how the natural/systemic productions of estrogens differs from that of exogenous/synthetic production is key. Aside from the neuroprotective effects of estrogens, they exert many biological effects as well. Estrogens are mostly widely known for their sex-specific effects related to sexual determination and differentiation (54). Both genomic and non-genomic signal transduction pathways of estrogens alter the regulation of the cell cycle, this includes, but is not limited to proliferation and differentiation, but specifically cyclines and cycline-2dependent kinases (55–57). As a result of this effect on cell growth and cell progression, estrogens have direct cancerogenic effects and damage DNA and cellular proteins via highly reactive oxygen reactivity (55, 58). Thus, understanding and parsing out the hormonal effects from neuroprotective actions of estrogens are key to their use as therapeutics.

## Formation of Estrogens

**Figure 1:**
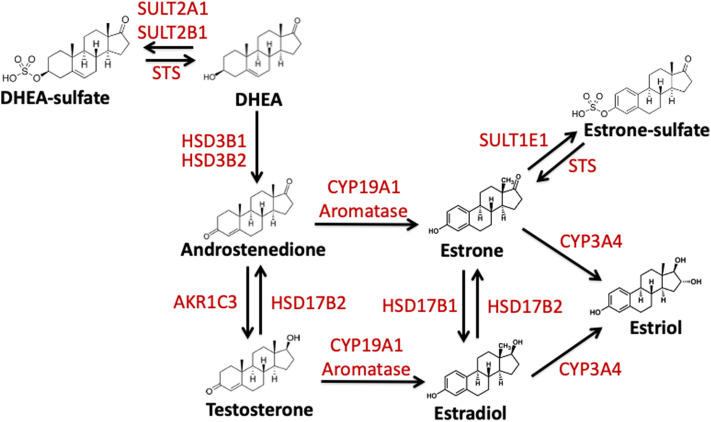
Schematic representation of the enzymatic conversion and synthesis of biologically active estrogens. Estrogens are produced from C19 steroid precursors through several enzymatic conversions. DHEA, dehydroepiandrosterone. DHEA-S, dehydroepiandrosterone-sulfate; Aromatase/CYP19A1, estrogen synthase; HSD3B1, hydroxysteroid 3 beta-1; HSD3B2, hydroxysteroid 3 beta-2; HSD17B1, hydroxysteroid 17-beta dehydrogenase; HSD17B2, hydroxysteroid 17-beta dehydrogenase 2; STS, steroid sulfatase; SULT2A1, Sulfotransferase Family 2A Member 1; SULT2B1, Sulfotransferase Family 2B Member 1; SULT1E1, estrogen sulfotransferase; AKR1C3, Aldo-Keto Reductase Family 1 Member C3; CYP3A4, Cytochrome P450 3A4.

Four estrogens are produced naturally: estrone (E1), 17β-estradiol (estradiol or E2), estriol (E3), and estetrol (E4; Figure 1) The weakest form estrone is primarily found following menopause while estriol and estetrol are the predominant estrogens produced during pregnancy (30). Weak estrogens can bind to estrogen receptors, but generally lack a dramatic effect within tissues or cells. However, some weak estrogens such as estriol exhibit greater protection than estradiol in autoimmune disorders, such as multiple sclerosis (59). Estradiol is the most common and strongest of the estrogens and is thought to mediate the neuroprotection following various damage to the nervous system (30). Estradiol is produced during the menstrual cycle and also *de novo* in the brain (60–62).

Estrogens are formed following the enzymatic conversion and interconversion from cholesterol-based precursors via a subset of enzymes termed estrogen-metabolizing enzymes (EME). The most prevalent of these enzymes is aromatase or CYP19A1. The aromatase pathway forms estrone and estradiol from androgenic precursors androstenedione and testosterone, respectively (Figure 1) (63). In addition to this estrogen synthase activity, aromatase has been proposed to regulate estrogen-2-hydroxylase activity in placental tissue and in Japanese quail brains (64–66). This activity also paired with aromatase's interaction with TH and DA signaling suggest that aromatase plays a role in catecholaminergic transmission (67, 68). Thus, aromatase may be involved in both the production and inactivation of estrogens (68). Another EME, 17β-hydroxysteroid dehydrogenases 1 and 2 (HSD17B1, HSD17B2) is also necessary for the conversion of estrone to estradiol (61, 69, 70). Finally, estrogens can be made inactive by both degradation and sulfonation. In the sulfatase pathway, inactive estrogen sulfate is the source or precursor for the active estradiol and estrone. This is mediated via the enzymes steroid sulfatase (STS) and estrogen sulfotransferase (SULT1E1) (Figure 1)(71, 72). Below I will review what is known about these EMEs and their role following TBI.

## EMES and TBI

### Aromatase

Among the EMEs, aromatase is the most prominent and widely studied. Across vertebrates aromatase expression is found in gonads, placenta, adipose tissue, bone, and other tissues including both male and female brains (73–75). Within the vertebrate brain, high concentrations of aromatase are expressed within the hypothalamus, amygdala, hippocampus, and cerebral cortex (76, 77). Aromatase is broadly expressed within neurons and not glial cells in the above listed brain areas of the uninjured brain (78–80). Aromatase is also present in pre-synaptic boutons, suggesting direct perisynaptic actions (81). Following injury or neuroinflammation, aromatase is also found in glial cells, specifically astrocytes (80, 82). In the songbird brain, females exhibit higher expression immediately after injury, but these differences disappear by 24 h post injury (83). This upregulated glial aromatase appears to affect neurodegenerative pathways by decreasing apoptosis (84, 85). In songbirds, as in the mammals (86), administration of fadrozole (aromatase inhibitor) dramatically increases the volume of damage induced by penetrating mechanical injury (84), sometimes in a sexually dimorphic manner (87). Replacing estradiol at the time of injury prevents this fadrozole-induced damage (88).

Cytokines increase aromatase expression without concurrent cell death or damage to neuronal tissues (25, 26, 89). Using TNF-α and IL-1β KO mice, we were able to determine that TNF-α, but not IL-1β signaling is necessary for the induction of aromatase following brain injury (25). Interestingly, while inflammation appears to regulate aromatase expression, increasing aromatase decreases expression of TNF-α and IL-1β following injury furthermore aromatase inhibition results in prolonged elevation of TNF-α and IL-1β (29, 89). Another mechanism by which estrogens may become inactive following TBI is through aromatase's estrogen-2-hydroxylase activity, that converts estrogens to catechol-estrogens (64–66). The role of this method of estrogen inactivation following TBI remains unknown. This cycle of both upregulation and inhibition of neuronal aromatase and cytokine expression may suggest a broadly conserved mechanism for protecting the CNS following detection of a threat (25).

### Steroid Sulfatase

In addition to the aromatase pathway described previously, estrogens can also be formed from inactive precursors by the removal of sulfate groups (90–93). When sulfated, estrogens are unable to bind and dimerize to estrogen receptors. This protects cells and tissues from excess estrogen activity (55). Thus, sulfonation can potentially regulate active estrogen signaling and serve as a hormone “reservoir” for future use (91, 94, 95). Steroid sulfatase (STS) hydrolyzes the removal of sulfate groups from estrone sulfate (E1-S) to E1 and dehydroepiandrosterone sulfate (DHEA-S) to dehydroepiandrosterone (DHEA), also known as androstenolone (Figure 1) (96). STS is expressed broadly across vertebrates in both males and females with highest levels being found in the placenta, but low levels found across the majority of steroid sensitive tissues. *STS* expression and the mechanism that control its expression remain poorly understood (97). However, estrogen signaling pathways regulate expression of STS, and thus potentially create a positive feedback loop to increase estrogen production and signaling (71).

The majority of studies examining STS expression and TBI have focused more on the beneficial effects DHEA vs. E2. As STS alters both estrogens and DHEA at this time we are unable to separate out the neuroprotective effects of estrogens vs. those of DHEA. Both DHEA and DHEA-S are neuroprotective following damage to the CNS in rodents and birds. Specifically, DHEA and DHEA-S protect rodent neurons against various forms of toxicity including overactivation of N-methyl-D-aspartate (NMDA) glutamate receptors (98, 99), amyloid beta expression (100), and oxygen-glucose deprivation (101). Importantly, DHEA-S prevents against rodent hippocampal neuronal cell loss and damage observed following ischemia and stroke (102). Additionally, the progesterone precursor pregnenolone and pregnenolone-sulfate also exhibit neuroprotective benefits (103–105). More work is necessary to discern estrogen mediated STS effects vs. DHEA mediated ones following TBI.

**Figure 2:**
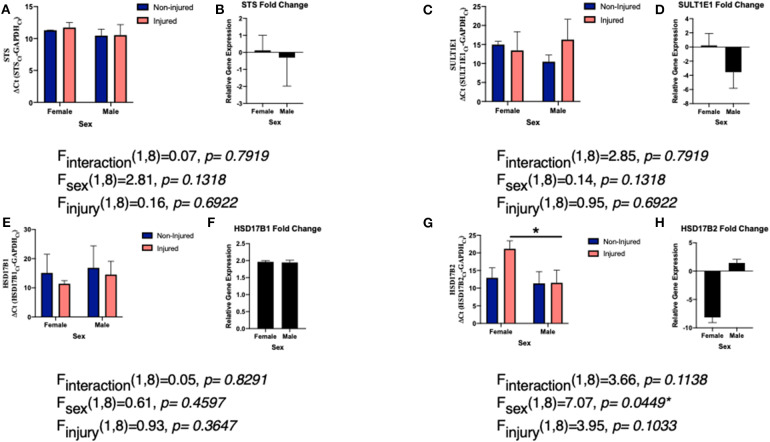
Levels of EMEs mRNA relative to glyceraldehyde-3-phosphate dehydrogenase (GAPDH; delta CT value and fold change) in both male and female zebra finches 24 h post-surgery. Adult male and female zebra finches were subjected to an unilateral penetrating injury directed toward the entopallium and collected 24 h later. Finches were sacrificed and total RNA was extracted from microdissections immediately adjacent to the cortical needle and the corresponding location on uninjured hemisphere. Expression of STS **(A)**, SULT1E1 **(C)**, HSD17B1 **(E)**, and HSD17B2 **(G)** relative to GAPDH was measured using quantitative PCR using primers specific for the ZF mRNA sequence. Mean ΔCT values were compared with a two-way ANOVA. Fold change in gene expression was also calculated STS **(B)**, SULT1E1 **(D)**, HSD17B1 **(F)**, and HSD17B2 **(H)**. Neither expression of STS, SULT1E1, or HSD17B1 were significantly changed following injury. However, HSD17B2 was significantly downregulated following injury. *denotes a significant difference at *P* < 0.05. All protocols were approved by the Vassar College Institutional Animal Care and Use Committee following National Institutes of Health Guidelines.

Like aromatase, we see a connection between STS expression and inflammatory signals. Interleukin (IL)-1β suppresses STS expression in endometrial stromal cells (106). However, in breast cancer cells, various other cytokines IL-1α, IL-6, TNFα, and enhanced or increased activity of STS (107). For both IL-6 and TNFα, STS mRNA expression was unchanged and the difference in activity was due to posttranslational modifications via STS glycosylation (72). Our studies examining the effects of a single penetrating injury in the adult zebra finch (*Taeniopygia guttata*) brain showed that STS expression was unchanged following TBI (Figures 2A,B). The zebra finch serves as a model organism for the study of steroid induced neuroprotection, because they rapidly and robustly respond to injury and express the full suite of steroidogenic enzymes (53, 82, 108). Furthermore, increased expression of pro-inflammatory cytokines is observed anywhere from 2 to 4 h following injury (83). Thus, the lack of a change in STS mRNA expression was surprising. As STS mRNA levels are unaffected in zebra finches, more research is necessary to determine if STS glycosylation or estrone bioavailability is increased following injury.

### Estrogen Steroid Sulfotransferases (SULT1E1)

SULT1E1 (Figure 1) when compared to other members of the steroid sulfotransferase (SULT) family has greater affinity for estrogens than other steroids, and thus readily inactivates estrone and estradiol. *SULT1E1* is expressed in various tissues in both sexes (109) including the brain (110) and its expression is regulated by steroid hormones, specifically progesterone (96). Estrone-sulfate and its naturally occurring counterpart estradiol-sulfate (E2-SO_4_) are biologically important as they regulate neuronal network formation, activity, and synaptogenesis (111–114) in vertebrates.

Very little is known about the expression of SULT1E1 following TBI, however there is some data on the role of estradiol-sulfate (E2-SO_4_) in rodent models. In terms of expression, like STS, we did not see a significant increase or decrease in expression of SULT1E1 following TBI (Figures 2C,D), however the expression was far more variable than that of STS. Studies are on-going to determine if protein expression matched the mRNA expression. Sulfated estrogens reduce neurodegeneration by altering cell and tissue damage and oxidative stress in rodents (115, 116). This is accomplished by improving blood and water flow to damaged tissue and thus increasing cerebral perfusion pressure and decreasing cell loss (22). Furthermore, the solubility of E2-SO_4_ as opposed to estradiol enables intravenous delivery of supraphysiological quantities and thus one could give much higher doses and have it be eliminated from the body much more quickly (117, 118). Sulfated estrogens provide a noteworthy avenue for both future research and future treatment options following TBI (118).

With respect to SULT1E1 and TBI induced inflammation, very little is known, but there appear to be a strong connection between SULT1E1 expression and release of cytokines. Knockdown of SULT1E1 increases pro-inflammatory cytokines and decreases anti-inflammatory signals (119). Furthermore, both edema and intercranial pressure are altered by SULT1E1 expression (115, 118). Understanding SULT1E1 expression and its role following TBI is key to understanding how sulfate estrogens can be used as a treatment. The time of SULT1E1 expression may also change the results. In the finch brain, we examined expression at 24 h, however the knockdown expression studies were 72 h. It would be interesting to extend the timepoints at which expression was examined.

### HSD17B1 and HSD17B2

17β-hydroxysteroid dehydrogenases (HSD17B) are enzymes responsible for the synthesis and deactivation of estrogens and androgens, specifically the formation of testosterone and estradiol from precursors (61, 76, 108, 120, 121) (Figure 1). HSD17B1 primarily catalyzes the conversion of estrone to estradiol in various tissues including the brain. In addition to its role in estrogen synthesis, HSD17B1 facilitates the formation of various androgens as well (76, 122–124). Conversely, the enzyme HSD17B2 mediates the oxidation of estradiol back to estrone, and testosterone and androstendiol back to androstenedione dehydroepiandrosterone. Furthermore, HSD17B2, is responsible for the production of the active progestin, progesterone (76, 125). The expression of HSD17B2 has previously only been identified in non-neuronal tissues, however a few reports have identified transcript and protein in the brain (126).

Surprisingly, there remains a dearth of information and studies on the role or expression of either HSD17B1 or HSD17B2 following TBI. The majority of past studies have examined the role of HSD17Bs in steroid sensitive cancers (69). Specifically, in terms of brain damage, there has been some work showing a connection between HSD17B1 and risk of Alzheimer's disease in Down Syndrome patients (127). In our work, we did not find a significant change in expression of HSD17B1 following TBI, suggesting that conversion to estrone is not a probable path to increasing estrogen signaling following injury (Figures 2E,F). While estradiol is the predominate estrogen mediating neuroprotection, estrone, has been shown to be neuroprotective following various damage or insults to the brain (128–130). Estrone increases the signaling of neuroprotective pathways (ERK1/2 and BDNF) and decreases cell death and thus ischemic injury size (38). These results suggest that despite the lack of change in HSD17B1, vertebrates may still be getting the protection from higher levels of estrone (38). As estrone is the most abundant estrogen in menopausal women (131, 132), it is hypothesized to be extremely important in mediating neuroprotection in this population (38). Much more work is necessary to understand the levels of estrone relative to estradiol following TBI.

While HSD17B1 expression was not altered by TBI, HSD17B2 expression was downregulated in a sex specific manner following TBI in the finch brain (Figures 2G,H). Females decreased expression of HSD17B2 following injury while males did not. Again, much remains unknown surrounding the function of HSD17B2 in the brain, specifically because it is noticeably absent from human and rodent brains (133). Notably other HSDs are found throughout the avian brain and regulate conversion from cholesterol to the sex steroids (108, 120, 134). Further research is needed in order to conclude if the expression of HSD17B2 following injury is unique to songbird brains, or if this represents a more evolutionarily conserved pathway. Despite the abundant questions remaining, the results are promising. One could hypothesize that females are decreasing expression of HSD17B2 in order to keep more estradiol available for neuroprotection and repair, while not having to produce more estrogens. As males use estrogen to undergo sexual differentiation, having a local upregulation of estrogens in the female brain may not be beneficial long term. Furthermore, while HSD17B2 is the key HSD17B isozyme in androgen and estrogen inactivation, it also activates 20α-hydroxyprogesterone into progesterone (70, 135). Females may be using progesterone in combination with estradiol as neuroprotective steroids following injury. These results are compelling as we have previously found a role for progesterone in cell survival following TBI in the brain (105) and this supports a large body of research examining progesterone treatment following TBI (136, 137). Interestingly, like with estradiol despite positive pre-clinical studies, larger clinical, and human trials of progesterone use following TBI have been unsuccessful (138). Together, these data suggest that much more research is needed in order to understand the very complex steroidal milieu following TBI.

## Conclusions

As has been discussed in the preceding sections, there is ample evidence to support that estrogens are neuroprotective following injury. Yet, when estrogen use has been used in clinical trials of TBI, the majority of evidence has not been positive and has led many to question its use as a treatment option (139, 140). Thus, more research on the formation of these estrogens and how they are inactivated is necessary in order to better develop treatment plans and options for mirroring estrogen's endogenous neuroprotective effects found preclinically without the undesirable hormonal ones observed.

## Ethics Statement

The animal study was reviewed and approved by Vassar College IACUC.

## Author Contributions

The author confirms being the sole contributor of this work and has approved it for publication.

## Conflict of Interest

The author declares that the research was conducted in the absence of any commercial or financial relationships that could be construed as a potential conflict of interest.
